# Higher estimated plasma volume status is associated with increased thrombotic risk and impaired survival in patients with primary myelofibrosis

**DOI:** 10.11613/BM.2023.020901

**Published:** 2023-04-15

**Authors:** Marko Lucijanic, Ivan Krecak, Ena Soric, Anica Sabljic, Davor Galusic, Hrvoje Holik, Vlatka Perisa, Martina Moric Peric, Ivan Zekanovic, Rajko Kusec

**Affiliations:** 1 Hematology Department, University Hospital Dubrava, Zagreb, Croatia; 2 University of Zagreb School of Medicine, Zagreb, Croatia; 3 Department of Internal Medicine, General Hospital Sibenik, Sibenik, Croatia; 4 University of Rijeka School of Medicine, Rijeka, Croatia; 5 Department of Hematology, University Hospital Center Split, Split, Croatia; 6 University of Split School of Medicine, Split, Croatia; 7 Department of Internal Medicine, “Dr. Josip Bencevic” General Hospital, Slavonski Brod, Croatia; 8 University of Osijek Faculty of Medicine, Osijek, Croatia; 9 Department of Hematology, Osijek University Hospital, Osijek, Croatia; 10 Department of Internal Medicine, General Hospital Zadar, Zadar, Croatia

**Keywords:** cancer, cardiovascular diseases, haematology, blood plasma, prognosis

## Abstract

**Introduction:**

Blood plasma represents a large reservoir of cytokines and other mediators of inflammation. Higher estimated plasma volume status (ePVS) has been shown to correlate with increased thrombotic risk in polycythemia vera patients, but its clinical and prognostic associations in patients with myelofibrosis are unknown which we aim to evaluate in this study.

**Materials and methods:**

We retrospectively analysed a multicentric cohort of 238 patients with primary (PMF) and secondary myelofibrosis (SMF). Estimated plasma volume status was calculated using the Strauss-derived Duarte formula. Overall survival (OS) and time to thrombosis (TTT) considering both arterial and venous thromboses were primary endpoints of interest.

**Results:**

Median ePVS was 5.8 dL/g and it did not significantly differ between PMF and SMF patients. Patients with more advanced disease features, more pronounced inflammation and higher comorbidity burden had higher ePVS. Higher ePVS (> 5.6 dL/g) was associated with shorter OS in PMF (unadjusted hazard ratio, HR = 2.8, 95% confidence interval, CI (1.79-4.41), P < 0.001) and SMF (unadjusted HR = 2.55, 95% CI (1.1-5.71), P =0.025) and with shorter TTT in PMF (> 7 dL/g, unadjusted HR = 4.1, 95% CI (1.44-11.59), P = 0.009) patients. Associations with OS diminished in multivariate analyses after adjustments for the dynamic-international-prognostic-scoring-system (DIPSS) and myelofibrosis-secondary-to-PV-and ET-prognostic-model (MYSEC-PM), respectively. Association with TTT remained significant independently of JAK2 mutation, white blood cell count and chronic kidney disease.

**Conclusions:**

Myelofibrosis patients with more advanced disease features and more pronounced inflammation have higher ePVS, indicative of expanded plasma volume. Higher ePVS is associated with impaired survival in PMF and SMF and higher thrombotic risk in PMF patients.

## Introduction

Chronic myeloproliferative neoplasms (MPNs) include chronic myelogenous leukemia (CML), defined by the presence of the Philadelphia chromosome, and three main Philadelphia chromosome negative (Ph neg) MPN clinical conditions: polycythemia vera (PV), essential thrombocythemia (ET) and primary myelofibrosis (PMF). Chronic thromboinflammation driven by constitutional activation of Janus kinase/signal transducer and activator of transcription (JAK-STAT) signaling pathway, caused by mutations in either JAK2, Calreticulin (CALR) or myeloproliferative leukemia virus oncogene (MPL) genes in majority of patients, is central to the pathogenesis of Ph neg MPNs (*1*). Both PV and ET patients suffer from high thrombotic risk and have a substantial risk of progression to secondary myelofibrosis (SMF), whereas PMF patients may experience a pre-fibrotic phase of the disease similar to ET but with a higher tendency for fibrotic and blast transformation (*2*). Patients with PMF and SMF have similar clinical presentation with the development of debilitating constitutional symptoms, anaemia and splenomegaly (*3*). However, due to a high risk of death and frequent disease-related symptoms, thrombotic risk is often overlooked in PMF and SMF patients and uncertainties still exist regarding the relevant risk factors and optimal prognostication.

Blood plasma represents a large reservoir of cytokines and other mediators of inflammation. Several recent studies have shown that increased estimated plasma volume status (ePVS) is associated with increased thrombotic risk in general population and in patients with established cardiovascular morbidity (*4*, *5*). Blood plasma experiences substantial changes in both volume and composition in patients with Ph negative MPNs. Higher ePVS has recently been shown to correlate with an increased thrombotic risk in PV, but its clinical and prognostic associations are unknown in patients with other MPN subsets (*6*). Thus, we aimed to evaluate clinical associations and prognostic significance of ePVS in a large multicentric cohort of patients with myelofibrosis.

## Materials and methods

### Study design

We retrospectively evaluated a cohort of 238 patients with PMF, post-PV and post-ET SMF diagnosed or referred to six haematologic centers in period from 2004 to 2021. Diagnoses were reassessed according to the 2016 World Health Organization (WHO) criteria for PMF and the 2008 International Working Group for Myelofibrosis Research and Treatment (IWG-MRT) criteria for SMF (*7*, *8*). Degree of bone marrow fibrosis was classified according to the current European consensus with grades 0 (scattered reticulin with no intersections), I (loose network of reticulin with many intersections), II (diffuse and dense reticulin, focal bundles of collagen, focal osteosclerosis) and III (coarse bundles of collagen with significant osteoclerosis). Risk stratification was performed according to the Dynamic International Prognostic Scoring System (DIPSS) in PMF and the Myelofibrosis Secondary to PV and ET-Prognostic Model (MYSEC-PM) in post-PV and post-ET SMF patients.

Estimated plasma volume status was calculated using the Strauss derived Duarte formula: 100-haematocrit (%)/haemoglobin (g/dL) and expressed as dL/g. Primary endpoints of interest were overall survival (OS) and time to thrombosis (TTT) considering both arterial and venous thrombotic events compositely (*5*). Deaths, arterial and venous thrombotic events and time to events were recorded from patient history. Arterial hypertension, diabetes mellitus, obesity, hyperlipoproteinemia and smoking were considered as cardiovascular risk factors. Cumulative burden of comorbidities was evaluated using the Charlson comorbidity index. For molecular analyses, deoxyribonucleic acid (DNA) was isolated from full blood by QIAamp DNA Blood Mini Kit (Qiagen, Hilden, Germany; ID 51104). JAK2 V617F mutation was assessed by allele-specific polymerase chain reaction (PCR), whereas CALR and MPL exon 10 mutations were screened by high–resolution melting dye assays and any sample sequence that deviated from normal was Sanger sequenced. Following complete blood count (CBC) and biochemistry parameters with corresponding units were assessed: white blood cell count (WBC, x10^9^/L), percentage of circulatory blasts, haemoglobin concentration (g/L), haematocrit (%), platelet count (x10^9^/L), lactate dehydrogenase (LD, U/L), C reactive protein (CRP, mg/L), albumin (g/L), ferritin (µg/L) and modification of diet in renal disease estimated glomerular filtration rate (MDRD eGFR, mL/min/1.73m^2^). Complete blood count was obtained using the Siemens Advia 2100, Siemens Advia 2120i (Siemens Medical Solutions Diagnostics Pte Ltd., Swords, Ireland), and Sysmex XN 1000 (Sysmex Europe GMBH, Norderstedt, Germany) analysers.

The study was approved by the Institutional Review Boards of University Hospital Dubrava (2020/0306-05), University Hospital Center Split (2181-147-01/06/M.S.-19-3), University Hospital Center Osijek (R2-1060/2020), General Hospital Zadar (02-2025/20-6/20), General Hospital of Šibenik-Knin County (01-3618/1-20) and Dr. Josip Benčević General Hospital (04000000/20-37).

Findings presented in the current paper have been previously presented as a poster on the European Haematology Association (EHA) congress 2022 and the Society of Haematologic Oncology (SOHO) congress 2022. Estimated plasma volume status was subsequently used as a predictor of thrombotic events in our subsequent work citing these findings (*3*).

### Statistical analysis

Normality of distribution of numerical variables was analysed using the Shapiro-Wilk test. Due to non-normal distribution, numerical variables were presented as median and interquartile range (IQR) and were compared between groups using the Mann Whitney U test and the Kruskal-Wallis ANOVA. Categorical variables were presented as frequencies and percentages and were compared between groups using the chi-squared test. Survival analyses were based on the Kaplan-Meier method. Time-to-event data between groups of patients were compared using the Cox-Mantel version of the log-rank test. Screening of survival associations was performed using the custom-made Microsoft Excel workbook (Microsoft, Leeds, United Kindom). The Cox regression analysis was used for multivariate analysis. The significance was set at P < 0.05. MedCalc Statistical Software version 20.109 (MedCalc Software Ltd, Ostend, Belgium) was used for all presented analyses.

## Results

There were a total of 238 patients analysed, 168 patients with PMF, 34 with post-PV SMF and 36 with post-ET SMF. Median age was 68 years, IQR (60-75). There were 147/238 (61.8%) males. Intermediate-2 or high-risk disease was present in 80/167 (47.9%) PMF and 30/61 (49.2%) SMF patients. Median ePVS was 5.8 dL/g, IQR (4.39-7.69). Estimated plasma volume status did not significantly differ regarding etiology of myelofibrosis (median 5.6 *vs* 5.9 dL/g in patients with PMF and SMF, respectively; P = 0.514).

**Figure 1:**
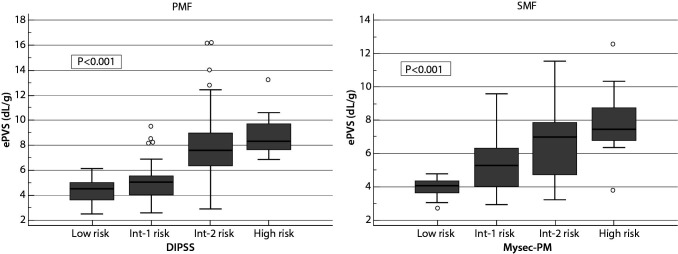
A) Unadjusted associations of estimated plasma volume status (ePVS) with dynamic international prognostic scoring system (DIPSS) risk categories in primary myelofibrosis (PMF), and B) myelofibrosis secondary to PV and ET prognostic model (MYSEC-PM) risk categories in secondary myelofibrosis (SMF) patients.

Patients’ characteristics and laboratory parameters stratified according to the ePVS are shown in Table 1 and Table 2, respectively (presenting unadjusted associations). In an overall cohort, higher ePVS was significantly associated with older age (P < 0.001), higher degree of bone marrow fibrosis (P < 0.001), absence of JAK2 mutation (P = 0.012), presence of MPL mutation (P = 0.048), presence of constitutional symptoms (P < 0.001), transfusion dependency (P < 0.001), lower WBC (P < 0.001), presence of circulatory blasts (P = 0.011), lower haemoglobin level (P < 0.001), lower platelets (P < 0.001), higher LD (P < 0.001), higher CRP (P < 0.001), lower albumin (P < 0.001), higher ferritin (P < 0.001) and higher comorbidity burden (Charlson comorbidity index, P < 0.001). Higher ePVS was significantly associated with larger palpable spleen size in PMF (P = 0.032) but not SMF patients (P = 1.000). Higher ePVS was significantly associated with higher risk disease in both PMF (DIPSS, P < 0.001, Figure 1A) and SMF patients (MYSEC-PM, P < 0.001, Figure 1B).

**Table 1:** Patients’ characteristics in an overall cohort stratified according to the estimated plasma volume status (ePSV; cut off at median > 5.8 dL/g)

	**ePVS ≤ 5.8 dL/g**	**ePVS > 5.8 dL/g**	**P**
Age (years)	65 (57.5-72.5)	70 (64-76.5)	< 0.001*
Sex Male Female	74/119 (62.2%) 45/119 (37.8%)	73/119 (61.3%) 46/119 (38.7%)	0.894
Myelofibrosis type PMF Post-PV SMF Post-ET SMF	86/119 (72.3%) 20/119 (16.8%) 13/119 (10.9%)	82/119 (68.9%) 16/119 (13.4%) 21/119 (17.6%)	0.297
BM fibrosis 0-I II-III	56/119 (47.1%) 63/119 (52.9%)	16/119 (13.4%) 103/119 (86.6%)	< 0.001*
JAK2 mutated	89/117 (76.1%)	75/112 (67%)	0.127^†^
CALR mutated	14/105 (13.3%)	7/87 (8%)	0.243
MPL mutated	0/102 (0%)	4/87 (4.6%)	0.390^†^
Constitutional symptoms	31/119 (26.1%)	72/119 (60.5%)	< 0.001*
Transfusion dependency	5/119 (4.2%)	54/119 (45.4%)	< 0.001*
Palpable spleen size (cm)	3 (0-8)	4 (0-10)	0.110
Charlson comorbidity index	2 (2-4)	4 (3-5)	< 0.001*
CV risk factors^ǂ^	73/109 (67%)	85/110 (77.3%)	0.089
History of thrombosis	20/119 (16.8%)	19/119 (16%)	0.861
DIPSS (PMF) Low risk Intermediate-1 risk Intermediate-2 risk High risk	25/85 47/85 13/85 0/85	1/82 14/82 53/82 14/82	< 0.001*
MYSEC-PM (SMF) Low risk Intermediate-1 risk Intermediate-2 risk High risk	9/30 15/30 5/30 1/30	0/31 7/31 10/31 14/31	< 0.001*
Data are presented as median (interquartile range) or proportions (percentage). BM fibrosis grading is based on the European consensus with grades 0 (scattered reticulin with no intersections), I (loose network of reticulin with many intersections), II (diffuse and dense reticulin, focal bundles of collagen, focal osteosclerosis) and III (coarse bundles of collagen with significant osteoclerosis). *P < 0.05 was considered statistically significant. ^†^statistically significant at P < 0.05 when ePVS is treated like numerical variable. ^ǂ^considered as present if any of classical cardiovascular risk factors were documented (arterial hypertension, diabetes mellitus, hyperlipoproteinemia, obesity, smoking) for a patient. ePVS - estimated plasma volume status. PMF - primary myelofibrosis. SMF - secondary myelofibrosis. PV - polycythemia vera. ET - essential thrombocythemia. BM - bone marrow. JAK2 - Janus kinase 2. CALR - Calreticulin. MPL - myeloproliferative leukemia virus oncogene. CV - cardiovascular. DIPSS - the Dynamic International Prognostic Scoring System. MYSEC-PM - the Myelofibrosis Secondary to PV and ET-Prognostic Model.			

**Table 2:** Laboratory parameters in an overall cohort stratified according to the estimated plasma volume status (ePSV; cut off at median > 5.8 dL/g)

	**ePVS ≤ 5.8 dL/g**	**ePVS > 5.8 dL/g**	**P**
WBC (x10^9^/L)	11.4 (9.25-18.55)	8.9 (4.95-15.9)	0.001*
Circulatory blasts ≥ 1%	35/119 (29.4%)	54/119 (45.4%)	0.011*
Haemoglobin (g/L)	134 (122-150)	93 (84-100)	< 0.001*
Platelets (x10^9^/L)	412 (281-648)	265 (143-531)	< 0.001*
LD (U/L)	394 (263-586)	484 (350-797)	< 0.001*
CRP (mg/L)	2.3 (1.1-6.6)	8.0 (3.5-18.9)	< 0.001*
Albumin (g/L)	45 (42-47)	40 (37-43)	< 0.001*
Ferritin (µg/L)	90.8 (42.0-174.0)	241.0 (79.0-591.0)	< 0.001*
MDRD eGFR < 60 mL/min/1.73m^2^	12/83	18/91	0.353
Data are presented as median (interquartile range) or proportions (percentage). *P < 0.05 was considered statistically significant. ePVS - estimated plasma volume status. WBC - white blood cell count. LD - lactate dehydrogenase. CRP - C reactive protein. MDRD eGFR - modification of diet in renal disease estimated glomerular filtration rate.			

**Figure 2:**
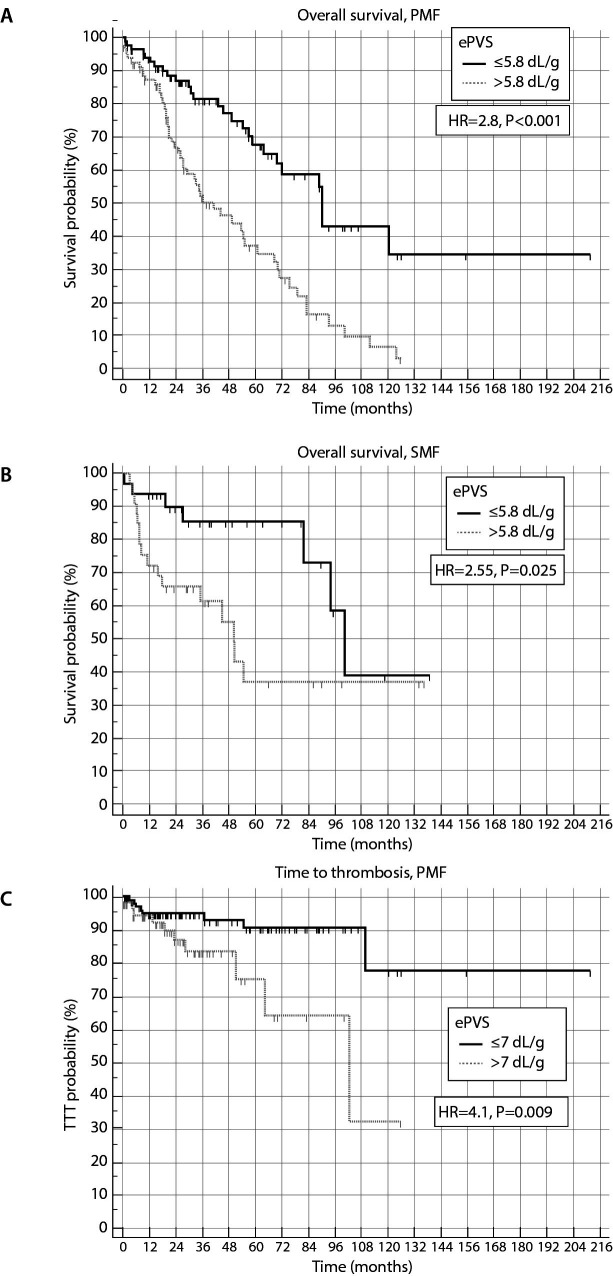
A) Unadjusted associations of estimated plasma volume status (ePVS) with overall survival in primary (PMF) and B) secondary myelofibrosis (SMF) patients, and with C) time to thrombosis in PMF patients.

Median follow-up of our cohort was 52 months. During follow-up period a total of 102 patients died, 28 patients experienced thrombotic event (26 arterial and 10 venous thrombotic events) and 16 patients experienced bleeding event. Higher ePSV stratified at median value (> 5.8 dL/g) was significantly associated with worse OS in an overall cohort of patients (unadjusted HR = 2.69, 95% confidence interval (CI) (1.81-4.0), P < 0.001), as well as among PMF (unadjusted HR = 2.8, 95% CI (1.79-4.41), P < 0.001, Figure 2A) and SMF patients (unadjusted HR = 2.55, 95% CI (1.1-5.71), P = 0.025, Figure 2B). Higher ePVS was significantly associated with shorter TTT in PMF (ePVS > 7 dL/g, HR = 4.1, 95% CI (1.44-11.59), P = 0.009, Figure 2C) but not SMF patients (P > 0.05).

We further evaluated independent prognostic properties of ePVS in a series of multivariate Cox regression analysis models. In separate PMF and SMF models investigating OS, adjusted for DIPSS and MYSEC-PM risk scores, respectively, associations of ePVS with OS diminished in both PMF and SMF patients whereas risk scores remained statistically significant in both analyses. In the multivariate Cox regression model investigating TTT in PMF patients, association of higher ePVS with increased thrombotic risk (adjusted HR 4.79, 95% CI (1.43-16.01), P = 0.011) was present independently of JAK2 mutational status (adjusted HR = 3.75, 95% CI (1.0-14.09), P = 0.049), WBC (adjusted HR 1.02, 95% CI (1.01-1.04), P = 0.002) and chronic kidney disease (adjusted HR = 3.56, 95% CI (1.29-9.8), P = 0.013), whereas cardiovascular risk-factors (adjusted HR = 6.37, 95% CI (0.76-53.22), P = 0.086) retained marginal significance adjusted additionally for age, sex, history of thrombosis and haematocrit.

## Discussion

The presented study is first to report that myelofibrosis patients with more advanced disease features and more pronounced inflammation have higher ePVS, indicative of an expanded plasma volume. Furthermore, higher ePVS was associated with an impaired survival in PMF and SMF and higher thrombotic risk in PMF patients.

Both PMF and SMF are diseases characterized by anaemia that may progress to transfusion dependency in a subset of patients. On the opposite, PV is characterized by high red blood cell count (cellular component of the blood). The importance of high blood viscosity for thrombotic risk prognostication in PV has been defined a decade ago (*9*). Nevertheless, except for the diagnostic purposes, scarce data currently exist on whether plasma volume (non-cellular component of the blood) may also have an impact on thrombotic and survival risks in Ph neg MPNs. This is probably due to the fact that measuring plasma volumes with the radioisotopes is cumbersome, expensive, toxic, and unavailable in most countries. However, a recent retrospective study in PV patients has shown that PV patients with higher ePVS may have a higher thrombotic risk (*6*). Considering the association of ePVS with cardiovascular morbidity in the general population and the fact that myelofibrosis patients suffer from high thrombotic risk, may have plasma expansion because of splenomegaly and anaemia (due to low haematocrit), we found it relevant to investigate the potential impact of ePVS on clinical characteristics and disease-related outcomes in patients with myelofibrosis (*4*, *10*).

Both disease-related (features of more advanced bone marrow fibrosis, cytopaenias, splenomegaly, stronger inflammatory drive) and unrelated factors (older age and higher comorbidity burden) seem to associate with higher ePVS in patients with myelofibrosis. Strauss-derived Duarte formula estimates plasma volume status by dividing the proportion of blood accounted by plasma (100-haematocrit) by haemoglobin concentration, thus making ePVS calculation proportional to the degree of anaemia (*5*). Transfusion dependency is common in patients with myelofibrosis due to untreatable cause of anaemia (bone marrow fibrosis) and both anaemia and transfusion dependency were associated with larger ePVS in our study. However, interestingly, there was no significant association of ePVS with presence of chronic kidney disease, especially when considering the role of kidneys in the plasma volume homeostasis. Chronic kidney disease is a potentially MPN-related phenomenon and recognized thrombotic predictor in MPN patients.

Most likely due to association of ePVS with anaemia, ePVS prognostic properties regarding survival (for which anaemia is an important prognostic determinant) are diminished after adjusting for DIPSS score. Nevertheless, prognostic properties of higher ePVS regarding thrombosis are present independently of other predictors of higher thrombotic risk in PMF patients like chronic kidney disease, presence of JAK2 mutation, and higher WBC. Understanding and refinement of thrombotic risk in myelofibrosis patients is of high importance as thrombotic events result in high degree of functional dependence and impose debilitating consequences on everyday life of these patients.

Main limitations of our work are retrospective study design and limited number of patients and events in specific subgroups to further characterize the clinical and prognostic role of ePVS in specific subsets of myelofibrosis patients. Also, no causal relationship can be inferred between investigated variables. Different CBC analysers were used in different institutions which may add to heterogeneity of the measured variables. Nevertheless, our multicentric study highlights the importance of blood plasma evaluation to better understand thrombotic risk in MPN patients. Future prospective studies on this very important topic are needed, hopefully answering the questions whether specific therapeutic approaches affecting plasma volume and composition may improve outcomes of myelofibrosis patients and whether non-invasive measures of blood plasma volume may guide clinical decision making.

## Data Availability

Data are available upon reasonable request.
